# Adherence to spinal imaging guidelines and utilization of lumbar spine diagnostic imaging for low back pain at a Canadian Chiropractic College: a historical clinical cohort study

**DOI:** 10.1186/s12998-022-00447-z

**Published:** 2022-09-16

**Authors:** Ali Smith, Varsha Kumar, Jeffrey Cooley, Carlo Ammendolia, Joyce Lee, Sheilah Hogg-Johnson, Silvano Mior

**Affiliations:** 1 Canadian Memorial Chiropractic College (CMCC), Toronto, Canada; 2 College of Science, Health, Engineering and Education, Murdoch University, Murdoch, WA Australia; 3 Rebecca MacDonald Center for Arthritis and Autoimmune Disease, Mount Sinai Hospital, Toronto, Canada; 4 Institute of Health Policy, Management and Evaluation, University of Toronto, Toronto, ON Canada; 5 Institute for Disability and Rehabilitation Research, Ontario Tech University and CMCC, Toronto, Canada; 6 Dalla Lana School of Public Health, University of Toronto, Toronto, ON Canada

**Keywords:** Low back pain, Radiographs, Chiropractic, Guidelines, Adherence, Red flags, Utilization rates

## Abstract

**Background:**

Diagnostic imaging is useful for assessing low back pain (LBP) when a clinician suspects a specific underlying pathology. Evidence-based imaging guidelines assist clinicians in appropriately determining the need for imaging when assessing LBP. A previous study reported high adherence to three clinical guidelines, with utilization rate of 12.3% in imaging of LBP patients attending a chiropractic teaching clinic. A new imaging guideline for spinal disorders has been published and used in teaching. Thus, the aims of our study were to assess the adherence to the new guideline and X-ray utilization in new episodes of LBP.

**Methods:**

We conducted a historical clinical cohort study using patient electronic health record audits at seven teaching clinics over a period of 20 months. Records of patients who were at least 18 years of age, presented with a new onset of LBP, and consented to data collection were included. Abstracted data included patient demographics, the number and type of red flags, and the decision to image. Rate of guideline adherence (proportion of those not recommended for imaging, given no red flags) and rate of image utilization were descriptively analyzed.

**Results:**

We included 498 patients in this study. At least 81% of included patients had one or more red flags reported. The most commonly reported individual red flag was age ≥ 50 (43.8%) followed by pain at rest (15.7%). In those referred for imaging, age ≥ 50 (93.3%) was the most frequently reported red flag. No red flag(s) were identified in 93 patient records, and none were referred for imaging of their LBP, yielding an adherence rate of 100% (95% CI 96, 100%). A total of 17 of 498 patients were recommended for imaging for their low back pain, resulting in an imaging utilization rate of 3.4% (95% CI 1.8, 5.0%).

**Conclusion:**

The imaging utilization rate was 3.4%, lower than 12.3% previously reported at a chiropractic teaching clinic. None without red flags were referred for imaging, yielding a 100%, adherence rate to current LBP imaging guidelines. Future research should consider currency of guideline, accuracy of red flags and factors influencing clinicians’ decision, when assessing imaging adherence rates.

## Introduction

Low back pain (LBP) is a major cause of global disease burden, with a lifetime prevalence estimated to be 80% in developed countries [1, 2]. Most LBP is non-specific and uncomplicated [1, 3–5]; however, pathology causing LBP, such as infection, malignancy, cauda equina syndrome and fracture account for less than 5% of cases in a primary care setting [1, 4, 6–10]. Evidence based guidelines recommend imaging a patient with LBP only if an underlying serious pathology is suspected, or if findings on the image could potentially result in a change in patient management [6, 7, 11, 12]. In the absence of these reasons, there is no added benefit to the patient, and unnecessary imaging can negatively affect patient outcomes, increase costs to the healthcare system, and expose the patient to unnecessary radiation [5–7, 12, 13].

Jenkins et al., reviewed the appropriateness of imaging in primary and emergency care settings and found 1/3 of patients were referred for imaging when there was no indication of underlying serious pathology suggesting over or inappropriate use [4]. Similarly, in Alberta, CN, of patients complaining of non-persistent LBP, 29.1% received an X-ray and 4.6% computed tomography (CT) or magnetic resonance imaging (MRI) [14]. Logan et al., reported that 16% of patients with signs and symptoms of non-specific low back pain were referred for CT examinations by Newfoundland, CN general practitioners [15]. Such variation in utilization is common within the Canadian health care system [14] and also evident in the chiropractic profession. Following a chiropractic consultation, X-ray utilization rates have been reported to range between 8 and 84% [6, 16], with almost 40% of chiropractors referring patients for imaging with non-specific low back pain [17]. In Ontario, CN almost 35% of patients with low back pain visiting a chiropractor between 1985 and 1991 received X-rays [18]; while at the chiropractic teaching institution, the utilization rate of imaging for a new episode of lumbar spine complaint was 12.3% in 2006 [8].

Guidelines have been developed to address this variation and potential inappropriate use of imaging; however, they too face variation in adherence to components of their recommendations [19]. For example, a 2014 best evidence synthesis suggests chiropractors demonstrated the highest level of overall adherence to acute and subacute LBP guidelines, but the lowest when assessing appropriate use of imaging in comparison to physiotherapists and medical practitioners [20]. Studies assessing adherence to imaging guidelines by chiropractors suggested rates ranging from 75% using clinical vignettes among private practice chiropractors in Newfoundland and Labrador, CN [10] to 89–94% for LBP at a Canadian chiropractic teaching institution [8]. Thus, the variation in adherence to clinical guidelines may impact the appropriate use of X-rays [4].

Clinical guidelines commonly recommend the use of red flag questions in the assessment of LBP when deciding to refer for imaging to rule out serious pathology. Despite this common recommendation, there is little evidence supporting their diagnostic accuracy [9, 21]; however, their presence or absence continues to be used as indicators for imaging [4]. For example, in assessing use of spinal imaging and adherence to guidelines, varying definitions involving red flags have been used, ranging from the absence of red flags to the presence of one or more and to only red flags attributed to serious pathology [2, 4, 8, 9, 22]. As a consequence, relying solely on red flags as indicators may lead to over- and under-use of imaging, and impact the potential of guidelines improving patient care [4].

In consideration of the latest spinal imaging guideline [11] and its implementation in current chiropractic curriculum at a Canadian teaching institution, we assessed if the guideline impacted the utilization rates of diagnostic imaging for LBP since last evaluated using previous guidelines [8]. The aims of our study were (1) to determine the adherence rate to the guideline and (2) the utilization of lumbar spine diagnostic imaging referrals in patients with a new episode of low back pain, presenting to a chiropractic teaching clinic.

## Methods

### Design and setting

We conducted a clinical historical cohort study at seven Greater Toronto Area (GTA) teaching clinics. Interns at these clinics are supervised by a licenced chiropractor and are responsible for working through history, physical examination, plan of management, and delivery of care. Clinical patient information is obtained and stored in the electronic health record (EHR) system.

### Recruitment and participants

Eligible patient records included were those of patients ≥ 18 years of age who presented with a new onset of LBP to one of the seven clinics. LBP was defined as pain or related symptoms (stiffness, aching or discomfort) located from the lower costal margin to the gluteal folds. A new onset of LBP was defined as LBP of less than 3 months in duration, preceded by a pain-free period of 4 consecutive weeks. Patients with concurrent back-related leg symptoms (numbness or pain) were also eligible. Records of patients who were chiropractic students, pregnant, or had already obtained any recent imaging (within the last 6 months) for their current episode of low back pain were excluded. Students were excluded from our study as some may have been attending the clinic for training purposes.

As part of the new patient intake forms at the Canadian Memorial Chiropractic College (CMCC), patients are provided with a privacy code describing the disclosure of personal information for research purposes if consent is granted. We included all eligible patients within the specified time frame who consented to the privacy code. This study was approved by the Research Ethics Board of the Canadian Memorial Chiropractic College (REB#192024).

### Data collection

We used electronic health records (EHR) to collect the data. Eligible records were identified consecutively using billing codes in the EHR between August 2018 and March 2020. To identify a new episode of LBP, billing codes for initial visit, re-evaluation and new complaint were obtained from the record. Only billing codes linked to a unique patient demographic number were included, thus preventing the same patient record from being included due to different complaints. Patients with student billing codes were excluded.

Each eligible patient record was assigned a unique sequentially generated numerical identifier. This unique identifier was linked to the patient file number and stored in a separate password protected file from the study data file. Each record was reviewed, and data were abstracted from the intake form, initial visit workup (history and physical), report of findings, and X-ray requisition form (if present). Patient records were reviewed from date of onset of new episode to a maximum of 7 weeks; this interval accounted for compliance with the recommended 4–6 week trial of care before requesting imaging [11]. In addition, clinical notes during this time interval were also reviewed to monitor any changes to the patient’s clinical presentation or changes to any red flags from the initial visit. We extracted the following variables from each patient record: age (yrs), sex, pain as per numerical rating scale (NRS) (numeric, 1–10), Oswestry Disability Index (ODI) score (numeric, 1–100), previous history of LBP (yes, no, uncertain), duration of symptoms (days), the presence of leg pain (yes, no, uncertain), location of pain (region), description of pain (text), diagnosis (text), if imaging obtained for current complaint (yes, no, uncertain), date of previous imaging for recurrent low back pain prior to this episode (date) and imaging modality (type).

Patients’ records were independently reviewed by a second investigator for study eligibility. All initial visits, new complaints and re-evaluations within a specified time frame were reviewed to ensure no patient was missed in the data extraction process from improper billing codes or those patients with LBP not receiving mobilizations or manipulations. Abstractors received standardized training on how to thoroughly audit patient files by members of the clinic management team at the chiropractic institution. Abstractors were provided definitions of red flags and key descriptors used in categorizing the presence of a red flag. Inter-rater reliability was used to assess agreement of the red flags between reviewers. This was calculated prior to any discussions between reviewers or weekly team meetings. To ensure data extraction was accurate, a co-investigator audited 40% of the patient files. During weekly team meetings, findings were reviewed, discussed and any coding disagreements were resolved by consensus, and so documented.

### Red flags

We extracted reported individual red flags in each record as described in the imaging guideline [11], and the data elements used to construct the red flags, along with which serious pathology they were affiliated with are presented in Table 1. If a red flag was reported in the patient record to be present, it was categorized as ‘yes’. If a red flag was reported as not present or negative, it was categorized as ‘no’. If there was insufficient information to determine the presence or absence of the red flag, it was categorized as “undetermined”. If a red flag was not documented in the file, it was categorized as “not reported”. A red flag was categorized as “not applicable” if considered to be unrelated to the patient (e.g., if a patient did not complete 4 weeks of care as the red flag was assessing worsening of symptoms for > 4 weeks).

**Table 1:** Individual elements extracted from the electronic health record to construct red flags

Individual elements	Serious pathologies				
	Neoplasm	Spinal fracture	Spinal infection	Inflammatory LBP	Sciatica*
Age ≥ 50	✓	✓	✓		✓
Pain at rest	✓				
Pain NRS (used to assess considerable pain)	✓	✓			
Pain minor trauma		✓			
Diabetic			✓		
Acute or blunt trauma		✓			
Hx of cancer (< 15 years)	✓				
Immunosuppressed due to condition			✓		
Improvement with activity, worse with rest				✓	
Known osteopenia or osteoporosis		✓			
Steroid use (> 7.5 mg/d, > 3 mo.)		✓			
No resp to tx or worse symptoms > 4 weeks	✓				✓
Hx of spine surgery			✓		
Morning stiffness > 30 min				✓	
Embedded Foreign object			✓		
IV drug abuse			✓		
Progressive neurological deficit > 4 weeks					✓
Significant activity limit > 4 weeks					✓
Awake 2nd half night due to pain				✓	
Fever (> 103°F/ > 38.3 °C) for 3 weeks			✓		
Progressive or painful spinal curvature		✓			
Unexplained weight loss (> 4.5 kg < 6 mo.)	✓				

*Suspected sciatica—lumbar disc herniation or degenerative spondylolisthesis or degenerative spinal stenosis

Finally, individual red flags were then grouped by common spinal pathologies as described in the imaging guideline [11] to assess referrals for imaging in the presence of red flag combinations as described in Table 2.

**Table 2:** Grouped red flags as per the spinal imaging guidelines [11]

Grouped red flags according to suspected serious pathologies
Neoplasm
History of Cancer (withing the last 15 years), Unexplained weight loss (> 4.5 kg over 6 months), Considerable LBP (NRS ≥ 8) and Age ≥ 50, failure of conservative care
History of cancer, failure of conservative care
History of cancer, considerable LBP and age ≥ 50
*Spinal fracture*
Recent significant trauma (any age)
Severe pain (NRS ≥ 8) with minor trauma, Age ≥ 50
History of prolonged corticosteroid use
Structural deformity
History of/high risk for osteoporosis
*Spinal infection*
Documented fever (> 38.3 °C) > 3wks, no established diagnosis, age ≥ 50
Diabetic
Immunosuppressed
Embedded Foreign object
IV drug abuse
*Inflammatory low back pain*
Morning stiffness > 30 min, improvement of pain with exercise but not rest, awakening 2nd half of night due to back pain
Any two of (morning stiffness > 30 min, improvement of pain with exercise but not rest, awakening 2nd half of night due to back pain)
*Suspected sciatica—lumbar disc herniation, spondylolisthesis, degenerative spinal stenosis*
Progressive neurological deficits with at least one of (age ≥ 50, failure of conservative care, significant activity limitations)

### Outcomes

We defined our primary measure of adherence as the proportion of patients not referred for lumbar spine imaging in the absence of any red flags as reported in the guideline [11] by interns and clinicians at the chiropractic institution. This measure was based on the previous imaging guideline adherence work by Ammendolia et al. [8], at the same teaching institution. We included secondary descriptive measures to explore the presence of red flags in those referred for imaging, namely (1) the proportion of patients referred for imaging with one or more red flags [4, 22], and (2) the proportion of patients with one or more red flags who were referred for imaging, and (3) the proportion of patients who presented with condition grouped red flags who were referred for imaging. We grouped red flags by condition as described in the guideline since the presence of a single red flag may not indicate the need for imaging [11], and because of considerable variation in the list and accuracy of red flags among different guidelines [9]. Referrals from external health care providers were documented in Table 5 and reported in our results but were not included in our analysis of adherence since the assessment of red flags and reason for imaging could not be confirmed. Finally, we estimated imaging utilization as the proportion of patients with a new episode of LBP who were referred for lumbar spine imaging by chiropractic interns and clinicians.

### Sample size

The planned sample size was based on achieving a width of confidence interval for a proportion of ± 5% for the primary adherence outcome, namely, the proportion of patients not referred for X-ray given no red flags. The width of the confidence interval for a proportion depends on the denominator, n, and also on the magnitude of the proportion, p, that is: normal approximation confidence interval for p is constructed as $\hat{p} \pm z_{\alpha } \sqrt {\hat{p}(1 - \hat{p})/n}$. The denominator for the primary adherence outcome, n, is the number of patients with no red flags. We considered a range of values for adherence from 0.85 to 0.99 and a range of values for proportion of patient files with no red flags from 0.3 to 0.55 based on previous literature, particularly the study by Ammendolia et al. [8] conducted in the same setting, where 30% to 55% of patients had no red flags and adherence ranged from 89.4 to 94.7%, depending on which guideline was considered. An overall sample size of 460 patients was found sufficient to achieve the desired width of confidence interval for these ranges of values.

### Analysis

Categorical variables and individual red flags were calculated using counts and percentages. Adherence outcomes were expressed as proportions with exact binomial 95% confidence intervals (95% CI). All statistical analyses were conducted using SAS software (SAS© 2018 SAS Institute Inc. SAS and all other SAS Institute Inc. product or service names are registered trademarks or trademarks of SAS Institute Inc., Cary, NC, USA).

## Results

**Fig. 1:**
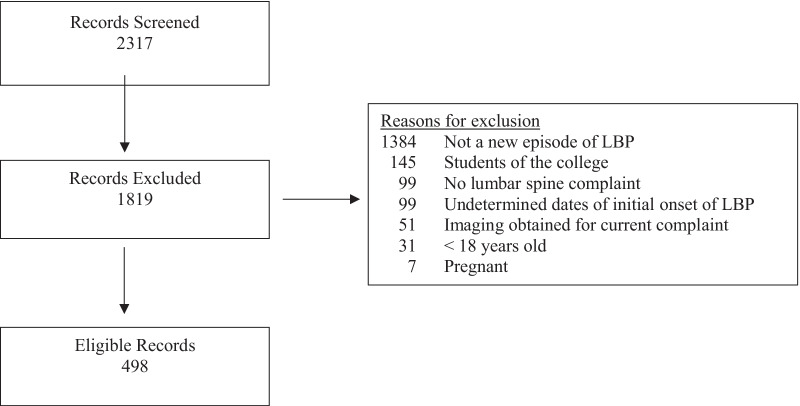
Summary of patient records

A total of 2317 patient records were screened, and 498 met the inclusion criteria (Fig. 1). The most frequent reason for record exclusion (n = 1384) was patients presenting with LBP of ≥ 3 months duration. A total of 201 files were audited which produced 4020 possible red flag variables. Of these there were 318 disagreements between data abstractors resulting in an inter-rater agreement of 92.1%.

The mean age of patients was 46.3 years of age and 45.4% were females. The majority reported a history of back pain with a mean duration of the current episode of about 19 days (SD 22.3). The average pain on a 10-point NRS was 5.4, with reported moderate level of disability. There were 62 (12.5%) patient records reporting reoccurring low back pain with previous imaging (> 6 months) for a prior episode of LBP. Only 17 (3.4%), 95% CI (1.8%, 5.0%) participants were referred for imaging for their current episode of LBP (Table 3).

**Table 3:** Patient recorded characteristics (N = 498)

Characteristic	Mean	SD
Age (years) (n = 498)	46.3	17.4
ODI score (n = 249)	40.0	18.8
NRS LBP (n = 435)	5.4	2.3
Duration (days) (n = 496)	19.4	22.3

		N (%)
Sex (n = 498)	Female	226 (45.4)
	Male	272 (54.6)
Associated leg pain (n = 491)	Yes	121 (24.6)
	No	370 (75.4)
Previous history of LBP (n = 377)	Yes	337 (89.4)
	No	40 (10.6)
Previous imaging for LBP (n = 107)	Yes	62 (57.9)
	No	45 (42.1)
Imaging requested (n = 498)	Yes	17 (3.4)
Utilization rate (%, 95% CI)		3.4% (1.8–5.0%)

ODI, Oswestry Disability Index; NRS, Numeric Rating Scale; LBP, Low back pain

No red flags were identified in 93 patient records (18.7%), 95% CI (78%, 85%), and none of these patients were referred for imaging. Therefore, based on our primary definition of adherence, the rate was 100%, 95% CI (96%, 100%).

We found 81.3%, 95% CI (78%, 85%) of patients’ records had one or more red flags reported. There were 20 different individual red flags identified as per the imaging clinical guideline, with varying frequency (Table 4). The most reported individual red flag was age ≥ 50 (43.8%), followed by pain at rest (15.7%), and considerable pain following minor trauma (9%). In those referred for imaging, age ≥ 50 (93.3%), pain at rest (53.3%) and considerable pain with minor trauma (26.7%) were most frequently reported. 100%, 95% CI (80.5%,100%) had at least one red flag present, while only 4.2%, 95% CI (2.5%, 6.6%) of the 405 patients with at least one red flag were referred for imaging. Of the 17 patients referred for imaging, 16 patients had two or more red flags present. In the single case where 1 red flag was present and imaging was requested, age ≥ 50 was the only recorded red flag. However, the patient reported worsening of symptoms and no response to care for three weeks.

**Table 4:** Presence and frequency of the individual Red Flags among records screened (N = 498)

Red Flags	Yes N [%]	No N [%]	UnD N [%]	NR N [%]	NA N [%]	Xray N = 15
Age ≥ 50	218 [43.8]	280 [56.2]	0 [0]	0 [0]	0 [0.0]	13
Pain at rest	78 [15.7]	113 [22.7]	43 [8.6]	263 [53.0]	0 [0.0]	8
Considerable pain (NRS ≥ 8) with minor trauma	45 [9.0]	346 [69.5]	1 [0.2]	106 [21.3]	0 [0.0]	4
Diabetic	45 [9.0]	441 [88.6]	1 [0.2]	11 [2.2]	0 [0.0]	3
Acute or blunt trauma	25 [5.0]	417 [83.8]	1 [0.2]	55 [11.0]	0 [0.0]	1
Hx of cancer (< 15 years)	23 [4.6]	448 [90.0]	1 [0.2]	26 [5.2]	0 [0.0]	2
Immunosuppressed due to condition	22 [4.4]	452 [91.0]	8 [1.6]	16 [3.2]	0 [0.0]	1
Improvement with activity, worse with rest	18 [3.6]	112 [22.5]	282 [56.6]	86 [17.3]	0 [0.0]	4
Known osteopenia or osteoporosis	16 [3.2]	0 [0.0]	0 [0]	482 [96.8]	0 [0.0]	2
Steroid use (> 7.5 mg/d, > 3 mo.)	14 [2.8]	456 [91.6]	5 [1.0]	23 [4.6]	0 [0.0]	0
No resp to tx or worse symptoms > 4 weeks	10 [2.0]	259 [52.0]	0 [0.0]	2 [0.4]	227 [45.6]	3
Hx of spine surgery	10 [2.0]	430 [86.4]	16 [3.2]	42 [8.4]	0 [0.0]	0
Morning stiffness > 30 min	8 [1.6]	53 [10.6]	40 [8]	396 [79.7]	0 [0.0]	0
Embedded Foreign Object	3 [0.6]	2 [0.4]	0 [0.0]	494 [99.2]	0 [0.0]	0
IV drug abuse	3 [0.6]	228 [45.8]	3 [0.6]	264 [53.0]	0 [0.0]	0
Progressive neurological deficit > 4 weeks	3 [0.6]	264 [53.0]	1 [0.2]	3 [0.6]	227 [45.6]	1
Significant activity limit > 4 weeks	3 [0.6]	262 [52.6]	0 [0.0]	2 [0.4]	231 [46.4]	1
Awake 2nd half night due to pain	2 [0.4]	170 [34.1]	63 [12.7]	263 [52.8]	0 [0.0]	0
Fever (> 103°F/ > 38.3 °C) for 3 weeks	2 [0.4]	103 [20.7]	5 [1.0]	388 [77.9]	0 [0.0]	1
Progressive or painful spinal curvature	2 [0.4]	10 [2.0]	2 [0.4]	484 [97.2]	0 [0.0]	0
Unexplained weight loss (> 4.5 kg < 6 mo.)	1 [0.2]	88 [17.7]	2 [0.4]	407 [81.3]	0 [0.0]	1

Hx, history; IV, intravenous; Resp, response; Tx, treatment; UnD, undetermined; NR, not reported; NA, not applicable

Table 5 displays the proportions of patients referred for imaging given the presence of red flag combinations as per the guideline [11]. When we grouped the red flags, 134 patients met at least one of the criteria based on combinations of red flags suggesting referral for imaging. No patient file met the 100% sensitivity criteria for neoplasm as reported by the guideline [11]. However, one patient had a history of cancer and failure of conservative care and was referred for imaging. Of the two patients with a history of cancer or considerable low back pain with age ≥ 50, only one was referred for imaging. Twenty-five patients had a history of significant spinal trauma at any age, and one patient was referred for imaging. Minor trauma with considerable low back pain and age ≥ 50 was identified in 19 patients, with 4 imaging referrals made. One patient had red flags suspicious of spinal infection as documented by fever and age ≥ 50 with no established diagnosis, who was referred for imaging. No patients were referred for imaging with red flags suggestive of inflammatory low back pain. All patients with suspected sciatica were referred for imaging. Four X-ray referrals were for patients not captured in Table 5; these had one or more red flags present (two were ≥ 50 with pain at rest, one was ≥ 50 with a history of cancer, and one was ≥ 50).

**Table 5:** Referral for imaging given presence of red flag combinations

Red flag criteria indicating imaging may be warranted	Meeting criteria N (% of 498)	Referred for imaging by chiropractor		Referred for imaging by other health care professional		Referred for imaging combined	
		#/N	% (95% CI)	# / N	% (95% CI)	# / N	% (95% CI)
*Neoplasm*							
History of cancer, unexplained weight loss, (considerable LBP and age 50 +), and failure of conservative care	0 (0.0%)	–		–		–	
History of cancer, failure of conservative care	1 (0.2%)	1/1	100 (3–100)	0/1	0 (0–97)	1/1	100 (3–100)
History of cancer, (considerable LBP and age 50 +)	2 (0.4%)	1/2	50 (1–99)	0/2	0 (0–84)	1/2	50 (1–99)
*Spinal fracture*							
Recent significant trauma (any age)	25 (5.0%)	1/25	4 (0–20)	0/25	0 (0–14)	1/25	4 (0–20)
Severe pain with minor trauma, age 50 +	19 (3.8%)	4/19	21 (6–46)	0/19	0 (0–18)	4/19	21 (6–46)
History of prolonged corticosteroid use	14 (2.8%)	0/14	0 (0–23)	1/14	7 (0–34)	1/14	7 (0–34)
Structural deformity	2 (0.4%)	0/2	0 (0–84)	0/2	0 (0–84)	0/2	0 (0–84)
History of/high risk for osteoporosis	16 (3.2%)	2/16	13 (2–38)	0/16	0 (0–21)	2/16	13 (2–38)
*Spinal infection*							
Documented fever > 3 weaks, no established diagnosis, Age 50 +	1 (0.2%)	1/1	100 (3–100)	0/1	0 (0–97)	1/1	100 (3–100)
Diabetic	45 (9.0%)	4/45	9 (2–21)	0/45	0 (0 -8)	4/45	9 (2–21)
Immunosuppressed	22 (4.4%)	1/22	5 (0–23)	0/22	0 (0–15)	1/22	5 (0–23)
Embedded Foreign object	2 (0.4%)	0/2	0 (0–84)	0/2	0 (0–84)	0/2	0 (0–84)
IV drug abuse	3 (0.6%)	0/3	0 (0–71)	0/3	0 (0–71)	0/3	0 (0–71)
*Inflammatory low back pain*							
Morning stiffness > 30 min, improvement of pain with exercise but not rest, awakening 2nd half of night due to back pain	0 (0.0%)	–		–		–	
Any two of (morning stiffness > 30 min, improvement of pain with exercise but not rest, awakening 2nd half of night due to back pain)	1 (0.2%)	0/1	0 (0–97)	0/1	0 (0–97)	0/1	0 (0–97)
*Suspected sciatica—lumbar disc herniation, spondylolisthesis, degenerative spinal stenosis*							
Progressive neurological deficits with at least one of (age 50 + , failure of conservative care, significant activity limitations)	3 (0.6%)	2/3	67 (9–99)	1/3	33 (1–91)	3/3	100 (30–100)

The utilization of imaging referral during the 20-month period of study was 3.4% 95% CI (1.8%, 5.0%). Fifteen patients were recommended for lumbar spine radiographs and two were recommended for MRI by CMCC clinicians/interns, with 66% of these referred after the initial visit. Of the 15 radiographic referrals, 14 were performed prior to study deadline. Degenerative changes without serious pathology were identified in 11 (78.6%) patients, and spinal fracture(s) in three (21.4%) patients. All patients with spinal fractures had 2 to 4 red flags consistent with spinal compression fracture as listed in the guideline [11] (Table 5).

Two patients were recommended for referral for MRI, rather than radiographic evaluation; one was obtained during the study timeline and demonstrated a lumbar disc herniation with corresponding nerve root compression. Seven additional imaging referrals were made from other health care professionals, of which three were for X-rays, three for MRIs and one for CT scan. Of these, the two referred for X-rays and three for MRIs were obtained within the study timeline, and no pathology was reported.

## Discussion

We found a 100% adherence rate to spinal imaging guidelines in patients experiencing a new episode of low back pain at a Canadian chiropractic teaching institution. We defined adherence as those without red flags who were not referred for imaging, similar to Ammendolia et al. [8], while appreciating the inconsistencies reported in the number and definition of red flags documented in guidelines across professions and in various countries [1, 9, 23, 24]. Our results compare favourably to previously reported adherence rates of 89–94% to three clinical imaging guidelines at the same teaching institution assessed 15 years earlier [8], and higher than the 75% adherence rate assessed using clinical vignettes among practicing chiropractors [10]. Parthipan et al., reported a 42% appropriateness to the same lumbar spine imaging guideline used in our study in an Australian chiropractic teaching institution [16]. However, their study did not analyze individual red flags; instead, their definition for appropriateness was based on the clinical information provided on the referral form and whether there was an indication for imaging (suspicion for underlying serious pathology, history of trauma, and contraindication to spinal manipulation) [16].

We found 81%, of patients had at least one red flag, which is similar to previous reports of 80–92.6% of patients presenting with at least one red flag to primary care settings [4, 6, 8, 9, 22, 25]. The highest reported red flag in our study was age ≥ 50, followed by pain at rest, which is different from a study evaluating red flags in primary, secondary and tertiary care settings that reported night pain as the most frequently reported red flag [25]. All patients who were imaged had at least one red flag, although only 4% of patients with any red flags were referred for imaging. This later proportion may suggest underutilization; however, current literature suggests red flags should not be considered in isolation but used in consideration of the entire clinical context of the patient [6, 8, 19, 23]. Instead, application of criteria considering sensitivity and specificity of red flags would be more appropriate [4], which was considered by Bussieres et al. when they grouped specific red flags by condition, as typically encountered in a clinical context [11].

Therefore, in consideration of the clinical context in which patients present, we grouped the red flags by condition as suggested by Bussieres et al. in the spinal imaging guideline [11]. In assessing grouped red flags in patients referred for imaging, we found conflicting results. Not all patients presenting with red flags consistent with grouped pathologies were referred for imaging, suggesting probable underutilization. This was noted in patients with red flags suggestive of serious pathology such as neoplasm, inflammatory LBP, and spinal fracture; however, none had such a diagnosis recorded in their EHR. Although the prevalence of serious pathology is very low, underutilization is a concern as missed pathology can lead to delayed diagnosis and treatment for patients. Jenkins et al., highlighted low quality evidence supporting inappropriate non-referral in 65.6% of cases when there were assessments of red flags, and 60% when decisions were made by clinical suspicion [4]. Alternatively, our clinicians and interns may have been making decisions based on the sensitivity and specificity of red flags given some isolated red flags are non-specific and may not all be indicators of true pathology (e.g., pain at rest or pain from minor trauma). Therefore, while red flags may have been present, taken within the context of the entire patient clinical picture, they may not have been concerning to the clinician/intern of significant pathology, thus imaging was not requested. We unfortunately did not directly consult with clinicians/interns to ascertain why patients with grouped red flags were or were not imaged; therefore, we cannot determine if this is true underutilization from improper guideline awareness or application. As new evidence emerges about the use of red flags in imaging, guidelines and curricula should be revised accordingly.

We found a 3.4% utilization rate for referral for imaging, which is lower than the previously reported 12% utilization at the same teaching institution [8] and the reported range between 8 and 33% for lumbar imaging utilization in other chiropractic teaching clinics [8, 16, 26]. Our utilization rate is also considerably lower than the 40% utilization rate reported in Canadian and American field chiropractors referring patients for radiographs with non-specific LBP [17], but similar to the 3% reported by French et al. in 2022 in a clinical trial aimed to reduce inappropriate imaging in primary care [19]. Possible explanations for the lower rate at our chiropractic institution include organizational emphasis and implementation of guidelines [6, 8, 19, 27, 28], teaching an evidence-based curriculum, discouraging routine and repeat radiographic examinations in the absence of red flags [6, 16, 28, 29], and potential under-use [4]. In contrast, chiropractors in private practice may be inclined to order imaging in patients with non-specific LBP due to perceived risks associated with treatment, fear of litigation, monitoring patients’ conditions and limit patient complaints [6, 7, 17]. In our study, 29% of patients referred for imaging (5 out of 17) had clinically significant pathology: 3 had confirmed spinal fractures, one had a degenerative spondylolisthesis, and one had a lumbar disc herniation. Assuming appropriateness of imaging and no underutilization, then this is slightly higher than a recent study from Australia that reported clinically significant pathology in 13% (19 out of 145 recommended for imaging) of those who had imaging obtained of their lumbar spine [16].

### Strengths and limitations

A strength of our study was that patient files were audited for a maximum to seven weeks to simulate a realistic course of care and enable monitoring of patient symptoms and response to treatment, including future decisions to image. Furthermore, an historical study design prevented any potential confounding bias from clinicians or interns changing their behaviour from a prior knowledge of the study. A limitation of our study was the challenges of interpreting the unique styles of clinical note taking in an EHR by interns/clinicians, and not clarifying ambiguous statements, thereby, possibly missing or miscoding red flags when extracting the data. However, in an effort to minimize potential misscoding, a second trained abstractor audited 40% of the patient files; however, we did not calculate kappa due to the extent of variables collected. We may also have misclassified or missed identifying eligible patients because of being limited to billing codes as opposed to identifying diagnostic codes in the EHR. Furthermore our study was conducted at a teaching institution and the results may not be generalizable to practicing chiropractors. Lastly, most red flags reported in the referent guideline are not standardized across other imaging guidelines or countries, no definitions were provided for the red flags (e.g. what constitutes “minor trauma”) nor the minimum number of red flags that suggest imaging should be obtained. Thus, we defined the red flags to ensure consistency throughout data extraction, and held weekly team meetings to review ambigious or complex files. It is possible during this process we interpreted patient symptomatology differently from what the guidline authors intended or the interpretation by the treating intern/clinician. We also are limited in identifying if true serious pathology was present in patients who were not referred for imaging. Finally, the referent guideline is dated and more recent research has questioned the accuracy of the red flags included in this guideline in identifying serious pathology [1, 2, 4, 23–25].

## Conclusion

We found no indication of lumbar spine imaging overutilization, as evidenced by a 100% adherence rate to LBP imaging guidelines in the absence of red flags. However, we did identify probable underutilization of imaging as assessed by red flags, both individually and when grouped by significant pathologies. Red flags should be continuously evaluated and interpreted within the context of patient history and presentation The radiographic utilization rate was 3.4%, which is lower than previously reported in the literature. Further research should consider currency of the imaging guideline, accuracy of red flags in identifying serious pathology and factors influencing clinicians’ decision when assessing adherence rate to spinal imaging in clinical practice.

## Data Availability

The datasets generated and/or analysed during the current study are available from the corresponding author on reasonable request.

## Abbreviations

LBP: Low back pain
EHR: Electronic health record
CT: Computed tomography
MRI: Magnetic resonance imaging
